# Investigation of species and environmental effects on rhubarb roots metabolome using ^1^H NMR combined with high performance thin layer chromatography

**DOI:** 10.1007/s11306-018-1421-1

**Published:** 2018-10-04

**Authors:** Yanhui Ge, Mengmeng Sun, Luis F. Salomé-Abarca, Mei Wang, Young Hae Choi

**Affiliations:** 1 Natural Products Laboratory, Institute of Biology, Leiden University, 2300 RA Leiden, The Netherlands; 2 School of Pharmacy, Xi’an Jiaotong University, Xi’an, 710061 China; 3 LU-European Center for Chinese Medicine, Sylviusweg 72, 2333 BE Leiden, The Netherlands; 4 Changchun University of Chinese Medicine, No. 1035, Boshuo Rd, Jingyue Economic Development District, Changchun, 130117 China; 5 SU Biomedicine, Postbus 546, 2300 AM Leiden, The Netherlands; 6 College of Pharmacy, Kyung Hee University, Seoul, 02447 Republic of Korea

**Keywords:** High performance thin layer liquid chromatography, NMR-based metabolomics, *Rheum palmatum*, *Rheum tanguticum*, Growth altitude

## Abstract

**Introduction:**

The pharmacological activities of medicinal plants are reported to be due to a wide range of metabolites, therein, the concentrations of which are greatly affected by many genetic and/or environmental factors. In this context, a metabolomics approach has been applied to reveal these relationships. The investigation of such complex networks that involve the correlation between multiple biotic and abiotic factors and the metabolome, requires the input of information acquired by more than one analytical platform. Thus, development of new metabolomics techniques or hyphenations is continuously needed.

**Objectives:**

Feasibility of high performance thin-layer chromatography (HPTLC) were investigated as a supplementary tool for medicinal plants metabolomics supporting ^1^H nuclear magnetic resonance (^1^H NMR) spectroscopy.

**Method:**

The overall metabolic difference of plant material collected from two species (*Rheum palmatum* and *Rheum tanguticum*) in different geographical locations and altitudes were analyzed by ^1^H NMR- and HPTLC-based metabolic profiling. Both NMR and HPTLC data were submitted to multivariate data analysis including principal component analysis and orthogonal partial least square analysis.

**Results:**

The NMR and HPTLC profiles showed that while chemical variations of rhubarb are in some degree affected by all the factors tested in this study, the most influential factor was altitude of growth. The metabolites responsible for altitude differentiation were chrysophanol, emodin and sennoside A, whereas aloe emodin, catechin, and rhein were the key species-specific markers.

**Conclusion:**

These results demonstrated the potential of HTPLC as a supporting tool for metabolomics due to its high profiling capacity of targeted metabolic groups and preparative capability.

**Electronic supplementary material:**

The online version of this article (10.1007/s11306-018-1421-1) contains supplementary material, which is available to authorized users.

## Introduction

Natural products are undeniably prolific sources of bioactive chemicals, constituting the basis of many fields of life sciences including drug development in pharmaceutics, functional foods, cosmetics agricultural products and industrial applications (Jeong et al. 2017; Newman 2016; Herrero et al. 2015). The original exploitation of plants as major sources of products for these applications has now been extended to a wide range of other natural products including microorganisms, insects and marine organisms.

Consequently, mankind has a long history of use of traditional medicine such as traditional Chinese medicine (TCM) and Ayurvedic medicine in India. However, in recent times, natural products are not often used directly but rather as a source of pure bioactive drugs. One of the reasons for abandoning the multicomponent herbal medicines may lie in the difficulty of controlling their quality and the appearance of unexplainable side-effects (Zhang et al. 2018; Arlt et al. 2002).

Recently, there is a new trend in natural products research, in which herbs or their derivatives are considered to be a bioactive product as a whole. This return to the use of plant extracts rather than the search of individual active compounds from the plants has been prompted by a number of reasons, such as the financially challenging process of obtaining a single active compound, the possibility of synergistic or potentiating effects of metabolites, and the recent great interest in personalized medicine (Stelzer et al. 2015; Wang et al. 2005). However, the widespread use of natural products can only be feasible if proper quality control methods based on comprehensive chemical profiling that covers the wide dynamic range of chemically diverse metabolites can be made readily available.

The rapid technological advances have allowed the development of a new trend in life science research known as systems biology a discipline that addresses scientific questions with a holistic approach. This requires new sets of tools and metabolomics is one of them. The goal of metabolomics is to detect and identify all metabolites involved in specific processes, a goal that cannot realistically be achieved by any one of the existing analytical methods. Therefore, the development of new analytical platforms is a hot issue in the metabolomics field. Solutions appear to come in the form of hyphenated techniques, often combining separation or extraction-based methods with spectroscopic analytical tools. Among these, the most well-known platforms combine NMR and MS with highly efficient chromatographic method such as LC (Wu et al. 2008; Mung and Li 2018; Porzel et al. 2014). However, despite their generalized use in the metabolomics field, there are a number of issues related to their application. For instance, the difficulty to integrate data, the lack of solutions for the identification of unknown metabolites, and increased measuring time are newly-generated problems.

In the active search for alternatives that can improve metabolomics data quality, the use of high performance thin layer chromatography (HPTLC), an efficient type of planar chromatography, was evaluated as a supplementary tool for metabolomics (Liu et al. 2018). Thin layer chromatography is one of the oldest chromatographic techniques and since its introduction has been used for chemical screening purposes due to its relatively short separation times, capacity for parallel analysis and broad range of metabolite detection either by direct visualization or chemical derivatization if necessary. Additionally, the development of highly efficient sorbents and the automation of critical TLC-steps, such as sample application, chromatographic development and detection have overcome many of the limitations of classical TLC analysis. Especially, this automation has a great impact on robustness of signals. Hence, all these improvements added to the ease of preparative scale work make HPTLC a useful supplementary tool for metabolic analysis (Ogegbo et al. 2012; Qian et al. 2007).

The feasibility and strength of HPTLC as a metabolomics platform, was evaluated in this study, using one of the most well-known medicinal plants species in TCM, roots of *Rheum* species (Radix et Rhizoma Rhei) as model plants. The influence of several factors such as genes (species) and environmental variations (geographical variation and collection altitude) on the metabolome were investigated.

Rhubarb is the dry root and root stock of several* Rheum* species, e.g. *Rheum palamtum* L., *R. tanguticum* Maxim. ex Balf. or *R. officinale* Bail (officially included in Chinese pharmacopoeia) as well as minor species such as *R. undulatum* L. (Lee et al. 2003). The most important constituents in rhubarb root are anthraquinone analogues such as chrysophanol, emodin, physicon, rhein, aloe emodin and their *O*- and *C*- glycosides (Aichner and Ganzera 2015; Pharmacopoeia of the People’s Republic of China 2005). A study on the correlation between chemical constituents and therapeutic effect of *Rheum* species showed that several anthraquinones like rhein and sennosides in *R. palmatum* appeared to be responsible for the purgative activity (Xiao et al. 1984). Another study stated that the sennoside content of rhubarb would suggest an association with high laxative activity, however the actual findings displayed low correlation between the total anthraquinone content and laxative activity (Oshio and Kawamura 1985).

Besides anthraquinones, various tannins (Kashiwada et al. 1986), chromones (Kashiwada et al. 1990) or stilbenoids (Yagi et al. 1971) were reported as minor compounds having other pharmacological activities. The content of anthraquinone derivatives was found to vary greatly among different *Rheum* species. For example, *R. palmatum* had a total content of 3.4%, while *R. tanguticum* contained only 1.2%. The emodin content in *R. palmatum* was higher than that of aloe emodin and *R. tanguticum* was found to contain a considerable amount of glycosylated rhein but no aloe emodin (Tang and Eisenbrand 1992, Su and Chen 1963). The tannin content also varied according to the species, ranging from approximately 11% in *R. palmatum* to 4–7% in *R. emodi, R. franzenbachii, R. hotaoense, R. officinale*, and *R. tanguticum*.

The variation in chemical composition was not limited to differences among species, and environmental conditions also showed a strong influence on the metabolites of rhubarb roots. The geographic location but even more so, the altitude proved to be a determining factor. For example, Sun et al. reported that the quality of *Rheum* roots was related to the elevation of the production area while their location had no effect whatsoever (Sun et al. 2016).

Despite extensive previous work, the detailed and overall correlation between quanti- and qualitative metabolite profiles, and biotic and abiotic factors is still unclear. In this study, this correlation was investigated using samples of two species of *Rheum* roots (*R. palmatum* and *R. tanguticum*) collected from five provinces in China with different altitudes, that were analysed by both ^1^H NMR and HPTLC. The data sets obtained with each method were firstly compared to evaluate their advantages and drawbacks. In view of the results, a method consisting in the hyphenation of NMR- and HPTLC-based profiling techniques was developed and optimized, providing a tool that can improve the quality of current metabolomics.

## Materials and methods

### Chemicals and reagents

Aloe emodin (**1**), catechin (**2**) chrysophanol (**3**), emodin (**4**), emodin-8-*O*-β-d-glucoside (**5**), physcion (**6**), rhein (**7**), sennoside A (**8**) and sennoside B (**9**) were purchased from Chengdu Pufei De Biotech Ltd. (Chengdu, China); the purity of all reference compounds was > 98%. Methanol, acetone, ethyl acetate, n-propanol (n-PrOH), cyclohexane and formic acid of analytical grade were purchased from Sigma (St. Louis, MI, USA). Deionized water was used in the mobile phase for HPTLC. Silica gel 60 F_254_ HPTLC plates (20 × 10 cm) were purchased from Merck (Darmstadt, Germany).

### Plant materials and sample preparation for analysis

The roots of *R. palmatum* L. and *R. tanguticum* (Maximowicz ex Regel) Maximowicz ex Balfour, Trans (125 samples in total: 55 for *R. palmatum* and 70 for *R. tanguticum*) were collected from five provinces of China: Gansu, Ningxia, Qinghai, Shaanxi and Sichuan in July–August in 2013; from diverse altitudes, ranging from 1441 to 4265 m above sea-level. The collected samples were air-dried for 3 days. The detailed information including voucher specimen numbers, altitude, latitude and longitude of the collection site as well as city and provinces of the samples employed in this study is listed in Table S1 (Supplementary material 2). The samples were identified by one of the authors, Mei Wang. Voucher specimens are deposited at the Natural Product Lab, Institute Biology of Leiden, Leiden University, Leiden, The Netherlands.

For NMR analysis, samples were treated using a slightly modified version of our previous protocol (Kim et al. 2010). Thirty milligram of each sample were vortexed with 1.0 ml of a mixture of KH_2_PO_4_ buffer (pH 6.0) in D_2_O containing either 5.8 mM of trimethyl silyl propionic acid sodium salt (w/w) (TMSP) and CH_3_OH-*d*_*4*_ (1:1) or 0.15 mM of hexamethyldisiloxane (HMDSO), then ultrasonicated for 20 min at 42 kHz and centrifuged at 13,000 rpm at room temperature for 5 min. An aliquot of 250 µL supernatant was transferred to a 3 mm NMR tube.

For HPTLC analysis, the same samples to ^1^H NMR analysis, dry powdered plant material (30 mg) was extracted with 1 mL of MeOH in a 2 mL-microtube and ultrasonicated at room temperature for 15 min. The extracts were then centrifuged at 13,000 rpm for 10 min. The supernatant was used for the analysis. A mixture of all the samples employed in this study was used as quality control (QC) samples.

### NMR analysis

^1^H-NMR spectra were recorded at 25 °C on a 600 MHz Bruker DMX-600 spectrometer (Bruker, Karlsruhe, Germany) operating at a proton Larmor frequency of 600.13 MHz. CH_3_OH-*d*_*4*_ was used as the internal lock. ^1^H-NMR experimental parameters were the following: 64 scans requiring 5 min and 13 s acquisition time, 0.16 Hz/point, pulse width (PW) = 30° (11.3 µs) and relaxation delay (RD) = 1.5 s. FIDs were Fourier transformed with LB = 0.3 Hz. The resulting spectra were manually phased, baseline corrected and calibrated to CH_3_OH-*d*_4_ at 3.3 ppm, using TOPSPIN 3.2 software (Bruker BioSpin GmbH, Rheinstetten, Germany).

Two-dimensional NMR parameters of ^1^H–^1^H-correlated spectroscopy (COSY), J-resolved and heteronuclear multiple bond correlation (HMBC) have been described in our previous reports (Kim et al. 2010).

### HPTLC analysis

All the samples were applied as 6 mm bands on HPTLC silica gel plates (60 F_254_), 20 × 10 cm (Merck) using a CAMAG automatic TLC sampler (ATS 4) (CAMAG, Muttenz, Switzerland) with a 25 µL Hamilton syringe. The samples were applied at 20.2 mm from the side edges and 10 mm from the bottom of the plate. The distance between samples was 11.4 mm and a total of 15 bands were applied per plate. The chromatographic development was carried out in an automatic developer (ADC2) (CAMAG, Muttenz, Switzerland). The samples were separated with three different mobile phases and conditions (1) *n*-PrOH–EtOAc–water (4:4:3, v/v/v), saturation time: 30 min, (2) EtOAc–MeOH–water (100:17:13, v/v/v), saturation time 25 min and (3) cyclohexane–EtOAc–MeOH–HCOOH–water (3:1:2:0.1:2, v/v/v/v/v, upper layer), saturation time: 30 min. For all chromatographic separations the humidity was set to 37% using a saturated MgCl_2_ solution. The solvent migration distance was 85 mm from the bottom of the plate. After development, images from all the plates were recorded with an automatic visualizer (CAMAG, Muttenz, Switzerland) under UV 366 nm. The HPTLC system was controlled by Vision Cats software. All plates were prepared similarly, spotting QC samples in the first three lanes and the plant extracts in the remaining lanes.

### Data processing for multivariate analysis

The ^1^H NMR spectra were automatically reduced to ASCII files. Spectral intensities were scaled to internal standard and reduced to integrated regions of equal width (0.04) corresponding to the region of δ 0.0–10.0 by AMIX software (Bruker). The regions of δ 4.7–5.0 and δ 3.28–3.34 were excluded from the analysis because of the residual signal of D_2_O and CD_3_OD, respectively.

The HPTLC data was processed using rTLC (version 1.0) according to the method developed by Fichou et al. (2016). Dimensions used to extract images were the same as those used for the sample application. The chromatographic tracks were bucketed every 128 units (pixel width), and parametric time-warping was applied to perform band alignment. For multivariate data analysis (MVDA), the grey channel data was used. After data extraction, all signals were normalized to the average of the QC samples intensity in each HPTLC plate.

Principal component analysis (PCA), partial least square (PLS) modeling, orthogonal partial least square (OPLS) modeling and soft independent modeling of class analogy (SIMCA) were performed with the SIMCA-P software (v.15, Umetrics, Umeå, Sweden). Unit variance (for HPTLC data) or Pareto (for ^1^H NMR data) scaling method was used both for PCA, SIMCA, PLS- and OPLS modeling. Permutation with 100 permutation cycles and CV-ANOVA were sued for the validation of PLS- and OPLS modeling.

## Results and discussion

### Overall metabolic profiling of the rhubarb samples by ^1^H NMR-based method

**Fig. 1:**
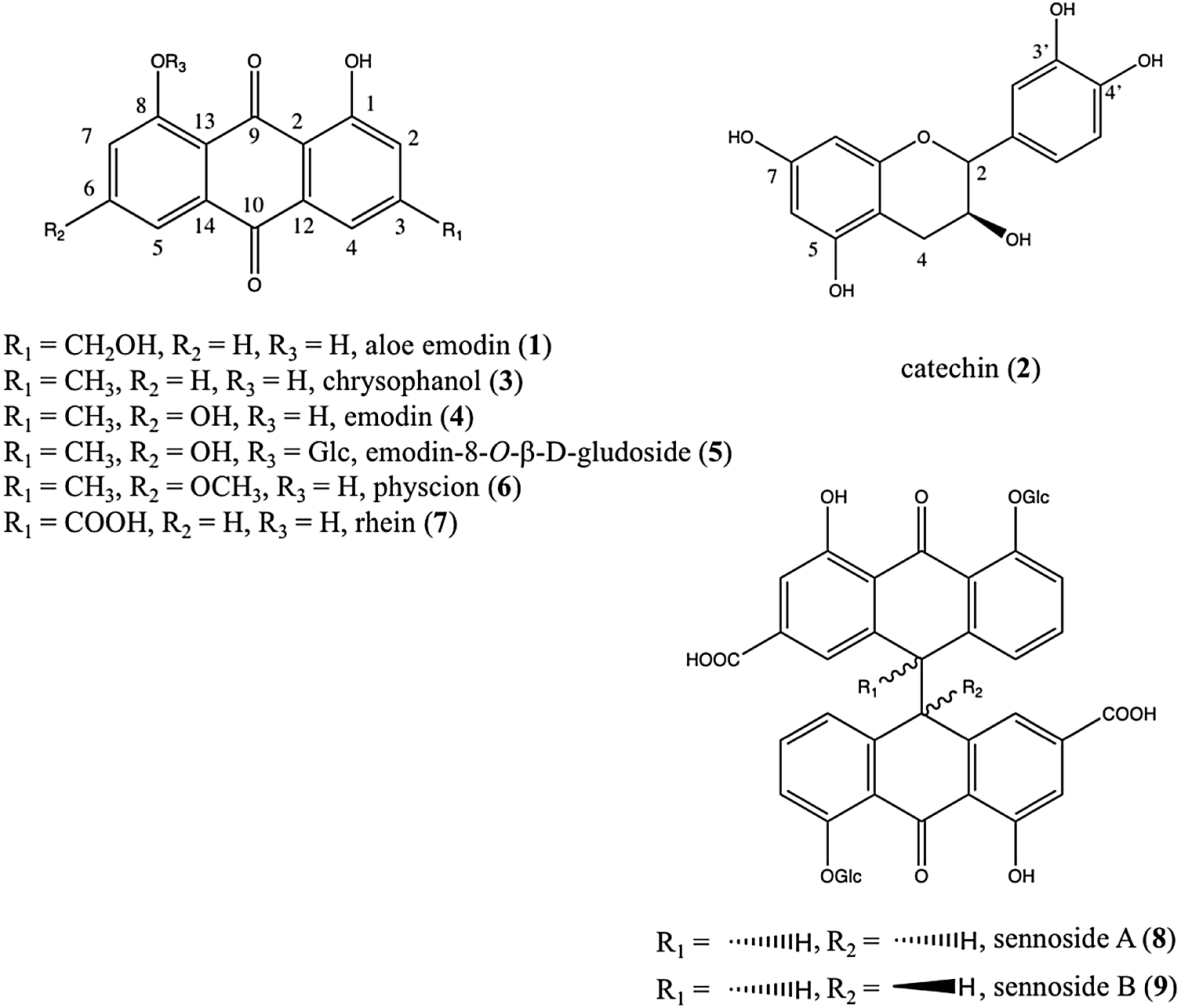
Chemical structures of eight anthraquinones and catechin used for the chemical profiling of *R. palamtum* and *R. tanguticum* roots

The ^1^H NMR metabolomics profiling was applied to the two *Rheum* species collected in different locations in order to profile a wide range of metabolites. In general, two solvents, MeOH or aqueous MeOH have been used as extraction solvents for plant metabolomics. Aqueous MeOH has proved to extract a higher number of hydrophilic primary metabolites while pure MeOH is usually more efficient for secondary metabolite extraction. This case was no exception and the major anthraquinones (Fig. 1) and catechin in *Rheum* species were much more soluble in MeOH than water-containing solvents. Except for emodin-8-*O*-β-d-glucoside (**5**), all the tested compounds were scarcely soluble in aqueous MeOH. In view of this, the rhubarb samples were extracted with CH_3_OH-*d*_*4*_ for further ^1^H NMR analysis including their multivariate data analysis.

**Fig. 2:**
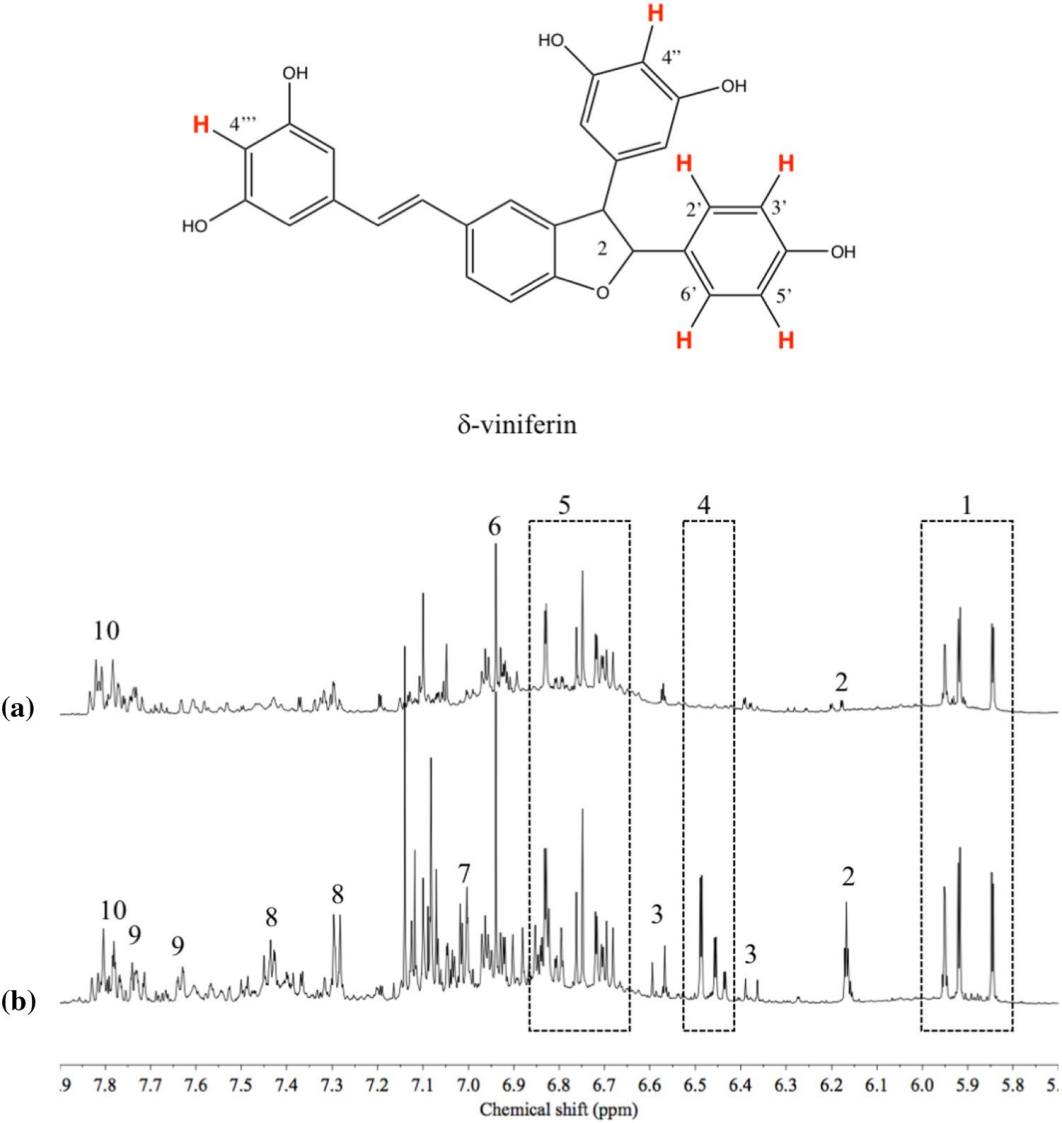
Typical ^1^H NMR spectra (600 MHz, CH_3_OH-*d*_*4*_) of *R. palmatum* (a) and *R. tanguticum* (b) roots. 1: H-6 and H-8 of catechin moieties of tannins, 2: H-4 of 1,3,5-trisubstituted benzene moieties in stilbenes, 3: H-8 of phenylpropanoids, 4: H-2 and H-6 of 1,3,5-trisubstituted benzene moieties in stilbenes, 5: H-2′, H-5′ and H-6′ of catechin moieties of tannins, 6: signals of gallica acid moieties, 7: H-3′ and H-5′ of 1,4-disubstituted benzene moieties in stilbenes, 8: H-2′ and H-6′ of stilbenes, 9: H-7 of phenylpropanoids, 10: signals of A and B rings of aloe emodin, chrysophanol rhein sennoside A and sennoside B

The ^1^H NMR spectra of the plant samples showed many primary metabolites including acetic acid, alanine, aspartic acid, asparagine, fumaric acid, glucose, lactic acid, sucrose and valine as well as lipids and phytosteroids (e.g. β-sitosterol). The characteristic metabolites were recognized in the aromatic region (Fig. 2). Among these, different types of tannins were well detected with signals at δ 5.8–δ 6.0 corresponding to the H-6 and H-8 of procyanidin type tannins containing catechin moieties and B-ring protons at δ 6.6–δ 6.9 of a catechin moiety including H-2′(d, J = 2.0 Hz), H-5′(d, J = 8.0 Hz) and H-6′(dd, J = 8.0, 2.0 Hz). The correction between the signals and splitting patterns were deduced from ^1^H-J-resolved and ^1^H-^1^H-COSY spectra.

Several stilbenes such as resveratrol (Wei et al. 2016) and δ-viniferin (maximol) (Shikishima et al. 2001) have been identified in *Rheum* species as minor compounds. The signal of H-4 of 1,3,5-trisubstituted benzene moiety at δ 6.17 (t, 2.2 Hz) and two signals of 1,4-disubstituted benzene moiety at δ 7.29 (H-2 and H-6, seemingly d, J = 8.3 Hz) and δ 7.01 (H-3 and H-5, seemingly d, J = 8.3 Hz) confirmed the presence of a stilbene (Fig. 2). These ^1^H resonances occur in a congested region and were assigned with the help of J-resolved and ^1^H-^1^H-COSY spectra and gallic acid moieties of *Rheum* tannins were clearly shown around δ 6.9 as a set of singlets.

In the case of anthraquinones, although they are major phenolic components of *Rheum* species, the signals were not clearly resolved due to the high congestion of the spectra.

To complete overall metabolic profiles of the sample set, ^1^H NMR data was analyzed further by multivariate data analysis. Firstly, PCA was carried out for the ^1^H NMR data. However, large chemical variations between the samples made it difficult to separate the plants according to their collection sites and altitude, or difference in species. None of the factors targeted in this study (species, collection sites and altitude) showed clear separation in the PCA score plot (Fig. S1 in Supplementary material 1).

**Fig. 3:**
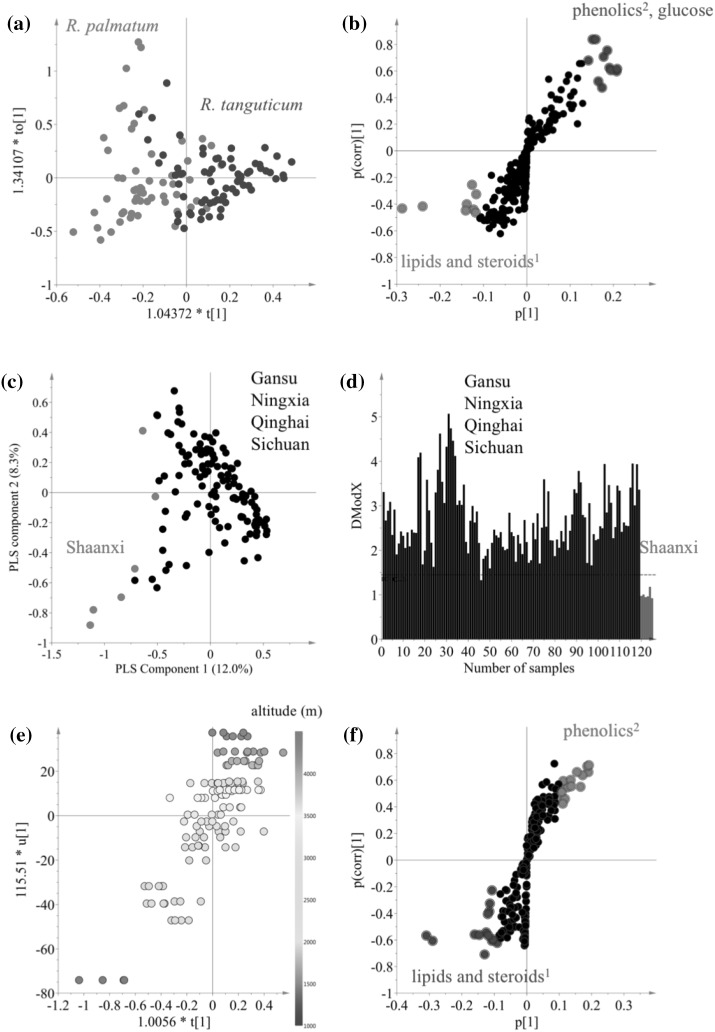
Multivariate data analysis of ^1^H NMR data of 127 *R. palmatum* and *R. tantuticum* roots. Score (**a**) and S-plot (**b**) plot of orthogonal partial least square modeling-discriminant analysis (OPLS-DA) using two species as classes. Score plot (**c**) of PLS-DA modeling using five collection provinces and DmodX plot (**d**) of SIMCA based on PCA-class for five collection provinces. Score (**e**) (t1/u1) and S-plot (**f**) plot of OPLS using altitude (m) of growing places. (1) Signals including δ 0.7–δ 1.6, δ 2.44, and δ 3.24, (2) signals including δ 6.0–δ 8.0

To confirm the correlation between metabolomic data and the factors, two supervised multivariate data analysis such as PLS and OPLS modeling, were applied to the same ^1^H NMR data set. Two of the three variables, species (*R. palamtum* and *R. tanguticum*) and collection provinces in China, (categorized as five provinces: Gansu, Ningxia and Qinghai, Shaanxi and Sichuan), were transformed to dummy variables for discriminant analysis. In the case of altitude, the data was used without any transformation. For multi-classes (e.g. collection provinces), PLS was used but for other factors, OPLS-DA or OPLS was employed. As shown in Fig. 3, all the factors tested in the study proved to have an effect on the metabolome of rhubarb roots to some degree and the models were validated by permutation test with 100 permutation cycles. *Rheum tanguticum* roots were well discriminated from *R. palmatum* roots (R^2^ = 0.58, Q^2^ = 0.45, *p* = 1.68e^−13^ in CV-ANOVA) due to their higher level of phenolics including anthraquinones, stilbenes and tannins (Fig. 3a, b). This data is not consistent with a previous report in 1963 by Su and Chen who reported that *R. palmatum* roots had almost three times the amount of anthraquinones than *R. tanguticum* roots (Su and Chen 1963, also referred to by; Tang and Eisenbrand 1992). This discrepancy might be not be such, since our results refer to the total content of phenolics of *R. tanguticum* roots, out of which anthraquinones are only one type of phenolic compound, while the specific content of anthraquinones in *R. palmatum* roots might be higher than in other species. Apart from this, the inconsistence in the results might be due to false-positive results obtained with the paper chromatography method used in that study by Su and Chen (1963).

The effect of collection sites was evaluated using, rhubarb samples grown in 28 cities in China that are well-known for the production of these plants. The collection site is not, per se, a scientific variable and more accurate metadata such as soil conditions, rain-falls, temperature, etc. should be used. However, in this case, the aim was not to investigate the influences of each individual environmental factor but rather to evaluate the significance of the variation between collection sites. Thus, the collection locations were categorized as five classes according to the provinces: Gansu, Ningxia, Qinghai and Sichuan. Firstly, the ^1^H NMR data corresponding to these five classes was analyzed by PLS-DA modeling. This resulted in the differentiation of samples from the Shaanxi province from all others (R^2^ = 0.75, Q^2^ = 0.46, *p* = 2.29e^−14^ in CV-ANOVA) as shown in Fig. 3c. To confirm this exceptional difference, SIMCA based on individual PCA of each class was performed and the distances between each class model were measured. As shown in Fig. 3d, both *R. palmatum* and *R. tanguticum* roots collected in Shaanxi province were significantly distinguished from other places. Using the loading plot of PLS-DA, the separation of Shaanxi samples was found to be due to a higher level of lipids and steroids and lower concentration of phenolics. However, before concluding that the roots from Shaanxi are exceptionally different, more samples from diverse locations in the other provinces should be analyzed. It is important to note that in this study, the number of samples collected in Shaanxi province was lower than those collected in other provinces.

One of the most influential factors on the metabolome of rhubarbs proved to be the altitude at which the plants were grown (Sun et al. 2016). All the samples employed in this study were collected in the range of 1441–4265 m. This altitude data was correlated with ^1^H NMR data by OPLS modeling. The model was validated using the permutation test (R^2^ = 0.70, Q^2^ = 0.58 and *p* = 0.43e^−19^ in CV-ANOVA). Figure 3e shows that the metabolites detected in the ^1^H NMR spectra are strongly correlated with the altitude (m) of their growing locations; PC1 (t1) of ^1^H NMR data is highly correlated with altitude (u1). The S-plot indicated that the metabolites associated with the altitude were phenolics including anthraquinones, stilbenes and tannins. This data is consistent with previous findings. Yan et al. found the contents of anthraquinones and tannins in rhubarb to increase significantly with increased altitude (Yan et al. 2017) and Sun et al. reported that the contents of anthraquinone derivatives and polyphenols in rhubarb were affected by altitude (Sun et al. 2016). This has been confirmed by other studies that concluded that environmental factors associated with altitude increased the phenol and flavonoid content in plants (Pandey et al. 2017). This was confirmed by Guerrero-Chavez’s research that revealed the role of altitude as a positive influence on phenolic synthesis (Guerrero-Chavez et al. 2015), possibly due to effects associated to an increase in altitude such as a gradual decrease of temperature and increase in light intensity.

In terms of the range of detected metabolites, ^1^H NMR showed a high profiling capacity for a broad range of metabolites including primary metabolites (amino acids, organic acids and sugars), and secondary metabolites such as anthraquinones, stilbenes and tannins, many of which could additionally be identified. The elucidation of anthraquinones which are known as main bioactive metabolites, however, was difficult since they were in the midst of a congested region of the spectra. Given their significance, this hindered the investigation of the influence of genetic and environmental factors on the *Rheum* metabolome. To circumvent the problems, HPTLC was applied to the same sample set, targeting on anthraquinone analogues (Fig. 1).

### Anthraquinone-targeted metabolic profiling of the rhubarb samples used a HTPLC-based method

Anthraquinones in *Rheum* species have a wide range of hydrophilicity depending on functional groups such as –OH and –CH_3_ as well as sugars in diverse positions. Three different HPTLC systems were used to detect the very diverse compounds: *n*-PrOH–EtOAc–water (4:4:3, v/v/v), EtOAc–MeOH–water (100:17:13, v/v/v), and cyclohexane–EtOAc–MeOH–HCOOH–water (3:1:2:0.1:2, v/v/v/v/v, upper layer).

**Fig. 4:**
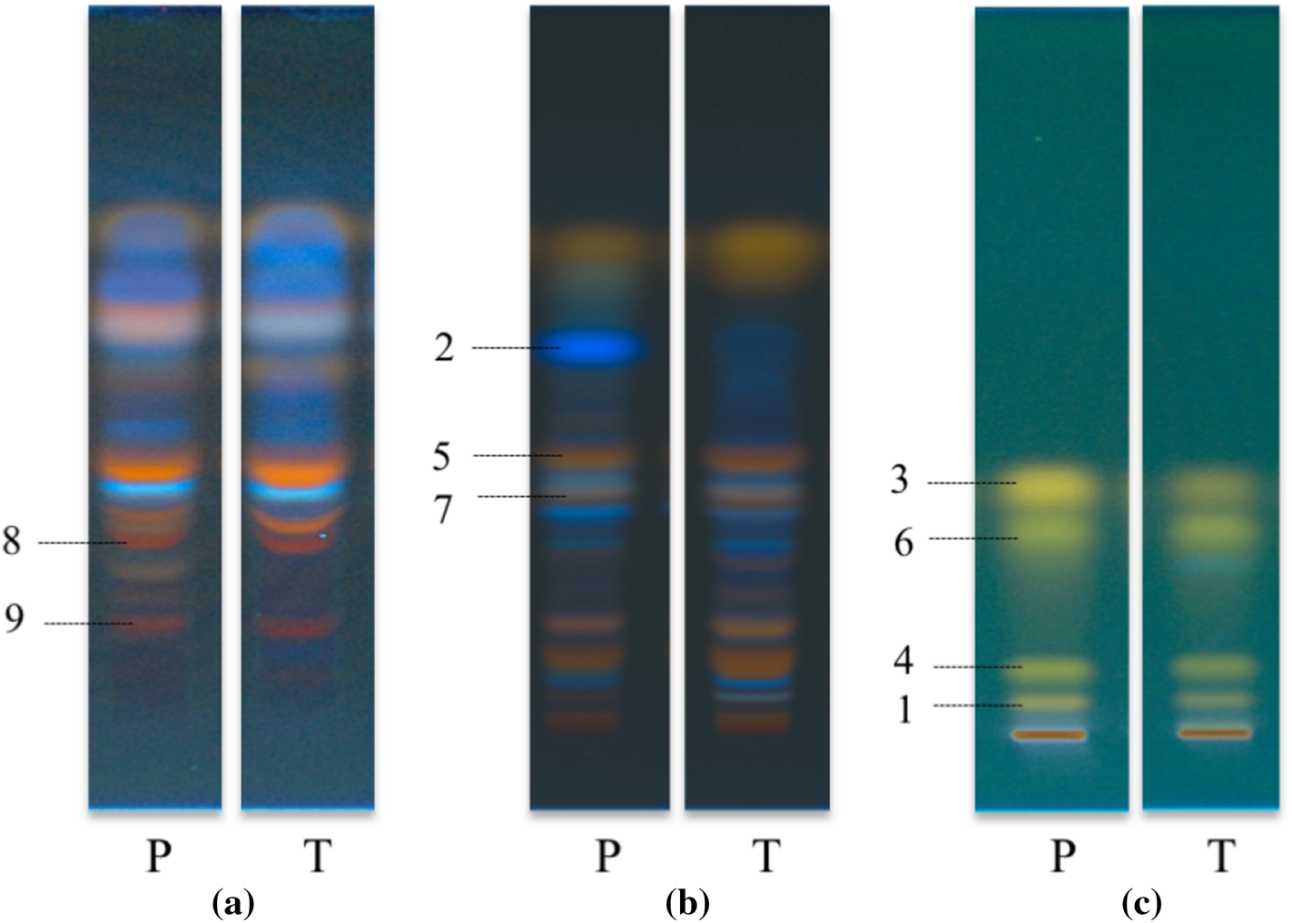
Typical high performance thin layer chromatograms of *R. palmatum* (p) and *R. tanguticum* (t) roots at 366 nm with different mobile phases. **a** *n*-propanol–EtOAC–water (4:4:3, v/v/v), **b** EtOAc–MeOH–water (100:17:3, v/v/v), **c** cyclohexane–EtOAC–MeOH–HCOOH–water (3:1:2:0.1:2, v/v/v/v/v, upper layer). 1: aloe emodin, 2: catechin, 3: chrysophanol, 4: emodin, 5: emodin-8-β-d-glucoside, 6: physcion, 7: rhein, 8: sennoside A, 9: sennoside B

Typical HPTLC chromatograms of *R. palmatum* and *R. tanguticum* roots obtained with the three mobile phases are shown in Fig. 4. The eight anthraquinones and catechin were well resolved with these systems.

The intensity of absorption at 366 nm of spots at all Rf values on the HPTLC chromatograms was measured using a visualizer. To reduce technical variations, the intensity of each spot (Rf) was normalized by the intensity of the corresponding Rf value in the QC samples. The normalized data was processed by rTLC (Fichou et al. 2016) for multivariate data analysis similarly to ^1^H NMR data, for which the data obtained from the three systems was combined to a single data matrix.

**Fig. 5:**
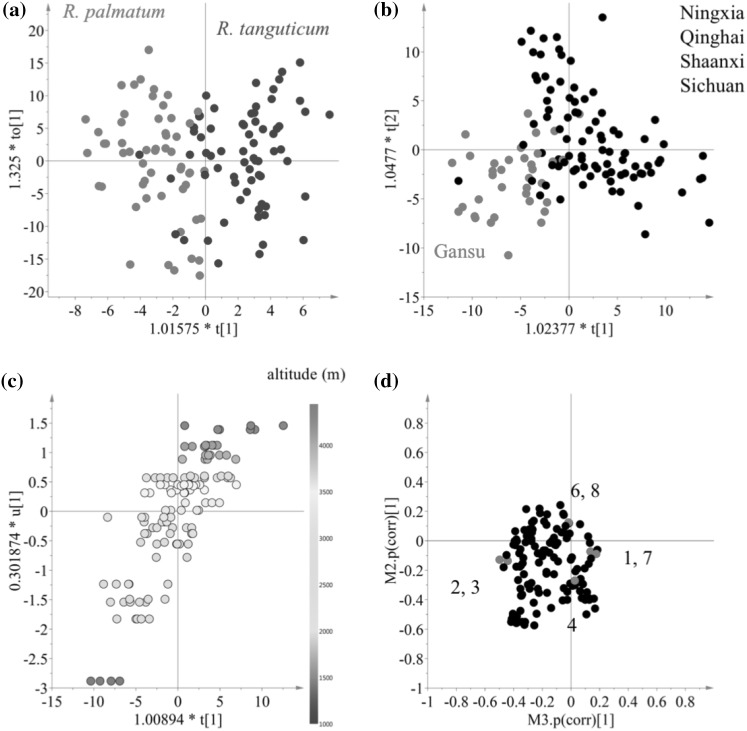
Multivariate data analysis of HPTLC combining data obtained with three mobile phases: *n*-propanol–EtOAC–water (4:4:3, v/v/v), EtOAc–MeOH–water (100:17:3, v/v/v) and cyclohexane–EtOAC–MeOH–HCOOH–water (3:1:2:0.1:2, v/v/v/v/v, upper layer). Score plots of OPLS-DA using two species (**a**) and five collection provinces (**b**) as classes. Score plot (**c**) (t1/u1) of orthogonal partial least square modeling (OPLS) using altitude (m) of growing sites. Shared-and-unique-structures (SUS)-plot (**d**) obtained from two combined OPLS models with the X-axis of species and Y-axis of altitude. The numbering in the SUS plot. 1: aloe emodin, 2: catechin, 3: chrysophanol, 4: emodin, 6: physcion, 7: rhein, 8: sennoside A

Similarly to ^1^H NMR data, clear separations were achieved with all the factors tested in this study such as species, collection locations and altitudes (Fig. 5). All the models were well validated by permutation with 100 permutation cycles and CV-ANOVA tests (R^2^ > 0.58, Q^2^ > 0.40 and *p* < 1.59e^−9^ in CV-ANOVA). Interestingly, while ^1^H NMR data, that covers a broad range of metabolites, led to the separation of samples collected in the Shaanxi province from all others, HPTLC analysis that targeted on anthraquinones from the same set of samples, resulted in the discrimination of samples collected in Gansu province (Fig. 5b). The analysis of the loading plot showed it might be due to in part to the relatively lower amount of sennoside A.

The correlation between metabolites and altitude were strongly correlated in HPTLC data as well as ^1^H NMR analysis. To deconvolute the individual metabolic relationship between species and altitude, a SUS-plot in which two OPLS models for species and altitude was used. As shown in Fig. 5d, higher levels of physcion (6) and sennoside A (8) were found in samples from higher altitudes while emodin (4) content was higher in samples from lower altitudes. As for the species-specific metabolites aloe emodin (1) and rhein (7) were found to be characteristic of *R. tanguticum* and catechin and chrysophanol of *R. palamtum*.

This coincides with previous reports on the species-specific nature of the anthraquinone derivatives content. For example, physcion and emodin have been reported to be the most abundant anthraquinone derivatives in *R. catharticus* while *R. orbiculatus* contained mostly physcion and chrysophanol (Locatelli et al. 2011) *R. alaternus* chrysophanol, emodin in *R. pumila*, and physcion in *R. fallax* and *R. intermedia* (Kosalec et al. 2013).

## Conclusions

There are many factors involved in the quality of the plants from which the medicinal drug rhubarb is obtained, and so far, no clear connection had been made between the presence of secondary metabolites such as anthraquinones and catechin and the quality. In the present study, a metabolomic study consisting in the multivariate analysis of data generated by HPTLC and NMR, was applied to develop an efficient tool for the quality control of the traditional Chinese medicine Rhubarb. The study revealed interesting and significant differences between the various species and altitude of growth. Whilst the NMR approach provided an easy quantification of the total anthraquinones contained in Rheum extracts as major components, the broad range of detection of HPTLC allowed the detection of metabolites that could not be identified by NMR. Results obtained with NMR-based metabolomics, showed that while both the species and sites of collection could influence the metabolite profile, altitude was the most discriminating factor, with anthraquinones, stilbenes and tannins being the important secondary metabolites that contributed to this separation. The HPTLC-based metabolomics study offered more information on the specific metabolites involved in the quality and influencing factors, showing that; physion, sennoside A and emodin were closely related to the altitude, while metabolites characteristic of each species were identified as aloe emodin and rhein for *R. tanguticum* and catechin and chrysophanol in *R. palamtum*. As anthraquinones are important metabolites for the therapeutic efficacy of Rheum species, it is essential to include strongly influential environmental factors as requirements to guarantee its metabolic content and resulting quality. This study shows the power of HPTLC as an analytical platform for a metabolomic study based on the Rf values and intensity of the chemotaxonomic markers, making a very valuable contribution to the quality control of rhubarb specifically but predictably to herbal drugs in general.

Although the resolution of HPTLC is not enough to do metabolic profiling by itself, it would be a promising supplementary tool when combined with other metabolomics tools such as NMR- or MS-based platforms.

## Electronic supplementary material

Below is the link to the electronic supplementary material.

Supplementary material 1 (TIFF 6774 KB)

Supplementary material 2 (XLSX 15 KB)
