# Supplementary figures and images for: Investigation of species and environmental effects on rhubarb roots metabolome using 1H NMR combined with high performance thin layer chromatography
Source: Metabolomics. 2018 Oct 4;14(10):137. doi: 10.1007/s11306-018-1421-1 (PMC6208752; doi:10.1007/s11306-018-1421-1)

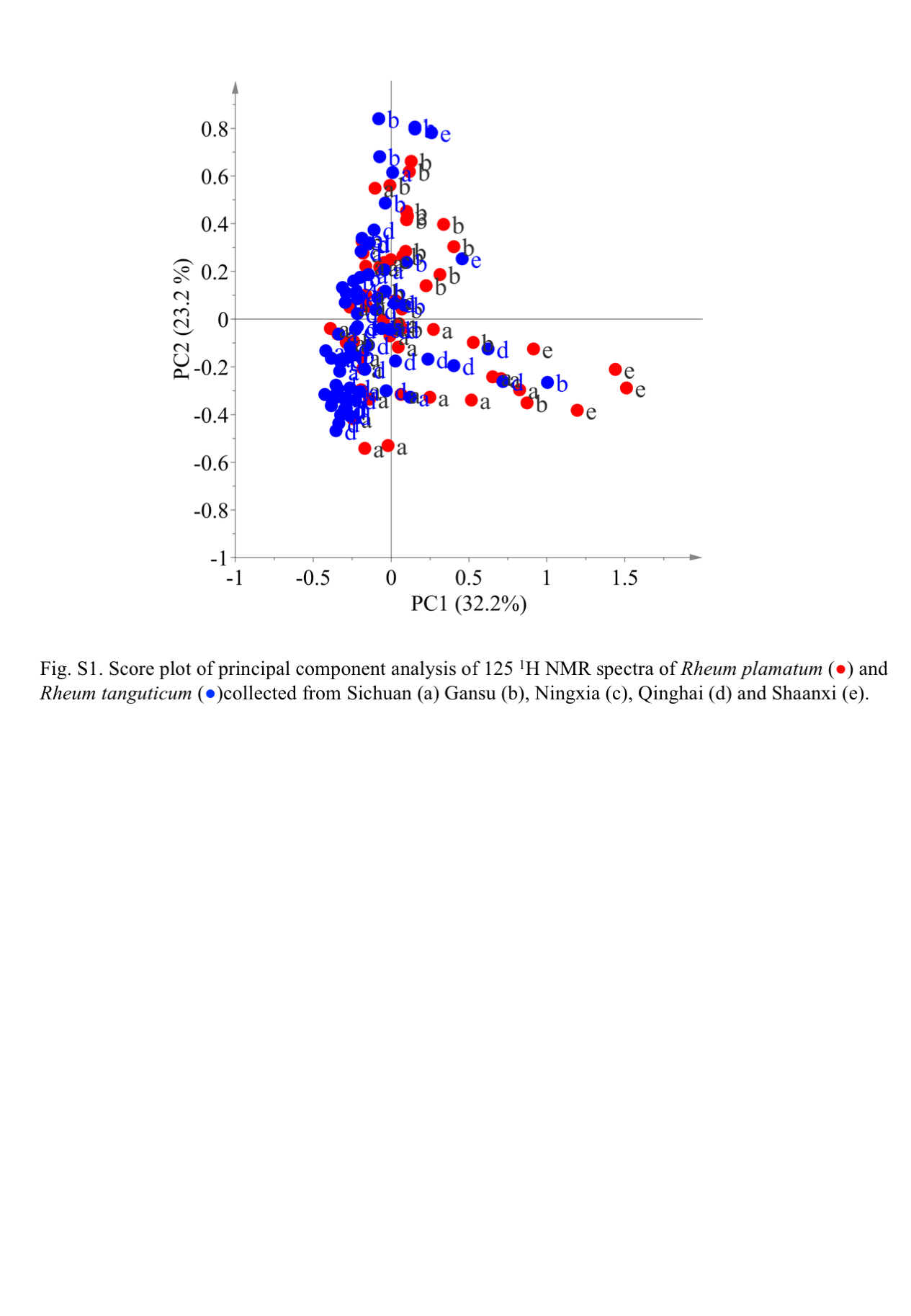

Supplement: Supplementary file 1 — Supplementary material 1 (TIFF 6774 KB) [file 11306_2018_1421_MOESM1_ESM.tiff]
